# Justice denied: reproductive injustices facing Black Queer birthing people in the U.S. healthcare system

**DOI:** 10.1186/s12978-025-02258-w

**Published:** 2026-01-24

**Authors:** Robyn B. Adams, Morgan E. Ellithorpe

**Affiliations:** 1 Department of Advertising & Brand Strategy, Texas Tech University, 2500 Broadway W, Lubbock, TX 79409 United States of America; 2 Department of Communication, University of Delaware, 246 Pearson Hall Newark, Delaware, 19716 United States of America

**Keywords:** Reproduction, Reproductive justice, Black, LGBTQIA+

## Abstract

**Background:**

This paper offers a critical review of reproductive injustices informed by secondary analysis of Black Queer birthing individuals’ narratives in the United States, focusing on how intersecting systems of oppression, particularly anti-Black racism, anti-queerness, and medical inequity, impact their access to and quality of reproductive health services, including assisted reproductive technology (ART), pregnancy, birthing, abortion, and postpartum care.

**Methods:**

Drawing on in-depth interviews with ten self-identified Black Queer birthing individuals from a prior published study (Qual Health Res 34:1039–52, 2024), we conducted secondary thematic analysis re-examining their narratives through the framework of obstetric racism, alongside extant health literature on medical racism, obstetric violence, and justice-oriented care to identify systemic barriers and areas where justice is routinely denied.

**Results:**

Findings revealed systemic barriers across access, support, and aftermath, with interview excerpts highlighting experiences of insurance discrimination, lack of bodily autonomy, and post-abortion mental health neglect. Participants often felt unheard, misgendered, or dismissed in clinical settings. Additionally, high ART costs, limited Black sperm donors, and inadequate mental health care exacerbated reproductive inequities. In contrast, community-based care models, such as those led by doulas and midwives, were mentioned by participants as affirming and empowering alternatives.

**Conclusion:**

Black Queer birthing individuals experience compounding systemic harms across the reproductive healthcare continuum. Addressing these injustices requires intersectional, justice-oriented healthcare reforms and stronger integration of community-based care models to ensure dignity, autonomy, and equity for all birthing people.

## Background

The Centers for Disease Control and Prevention (CDC) reports a decrease in pregnancy-related mortality rates for all racial/ethnic groups except Black birthing individuals (regardless of gender and/or sex identity) [1]. Specifically, Black Queer (including lesbian, gay, bisexual, non-binary, transgender (including non-binary trans)), queer, Two-Spirit, intersex, asexual, aromantic, pansexual, and questioning identities, among others) people face multiple forms of interconnected oppression, such as disproportionate rates of poverty, lack of health insurance, and unemployment [2], leading to a significantly higher risk of adverse pregnancy and abortion-related outcomes [3, 4]. Notably, Black birthing people (both cisheterosexual and queer) are losing their lives at a rate three times higher than white individuals due to inequitable healthcare rooted in anti-Black racism [1]. Furthermore, Black birthing people’s, including Black queer people’s, voices often go unheard in healthcare settings when sharing their health-related concerns and needs [3–5]. Most pregnancy- and abortion-related deaths among Black birthing people are preventable, underscoring the urgent need for systemic change [6, 7]. To address these issues, calls have been made for focused research on the reproductive healthcare experiences of Black birthing individuals, especially those specifically living with marginalized identities in sexuality, sexual orientation, sex and/or gender expression [3, 8, 9]. As a Black non-binary lesbian and reproductive justice activist [first author] and a White cishet woman scholar who has experienced pregnancy, childbirth, miscarriage, infertility, and assisted reproductive technology (ART) [second author], we propose recommendations for health disparities researchers and healthcare providers to better support Black Queer birthing people along their reproductive journeys and combat maternal mortality.

This paper builds on our previously-published study [3] that explored barriers Black Queer birthing individuals face along their gestational journeys within the healthcare system (HCS). While that paper presented the full narrative stories of the same ten participants, the current manuscript revisits those transcripts to examine previously underexplored patterns of obstetric racism and other systemic injustices shaping their reproductive-based HCS experiences. In particular, we focus on the research partners’ (or participants’) suggestions for how the system could do better in the future in empowering, rather than oppressing, Black Queer birthing people.

### Black birthing people’s experiences with reproductive healthcare

Research has shown that Black and Queer individuals face significant challenges in healthcare settings, including heightened discrimination (such as anti-Blackness and anti-queerness) and a lack of understanding from healthcare providers [3, 4, 10–12]. For instance, Black and Black Queer birthing individuals often feel that their healthcare providers are unprepared and emotionally unavailable when they seek support for 2SLGBTQIA+-centered reproductive health questions [3, 5, 13]. This situation puts Black and Black Queer birthing people at a greater risk of not receiving the necessary quality of care to ensure their well-being throughout their reproductive journeys. Despite expressing a desire for collaborative processes with their doctors, many Black Queer birthing individuals specifically feel unheard and dismissed when discussing their reproductive health needs, including birthing desires, pregnancy-related care, abortion support, and fertility services [3]. These stressful interactions with medical providers are compounded by the complexities of navigating healthcare insurance systems, leading to confusion and uncertainty regarding care. Black birthing individuals, regardless of their sexual orientation or gender and/or sex identity, who encounter issues with health insurance access often delay care or avoid medical expenses due to the stress involved in dealing with the insurance system [14]. Furthermore, research indicates that many gynecologists lack training in gender-affirming care and have limited education on 2SLGBTQIA+ -specific health issues [9]. With over 1.2 million Black individuals identifying as 2SLGBTQIA + in the United States, it is essential to consider the intersections of race, sexuality, and gender when addressing the Black maternal health crisis in the country [2]. A deeper understanding of how intersectionality affects the quality of care for Black birthing individuals is necessary to identify key intervention points. This knowledge can help improve reproductive health-related research and provider-patient communication by developing culturally sensitive and queer-affirming health training and resources grounded in a justice-oriented approach.

### Justice-oriented approaches to reproductive healthcare for Black Queer birthing people

Guided by Black feminist thought and reproductive justice frameworks articulated in our previous analysis [3], we approached the secondary analysis with the focus on obstetric racism. While the original analysis emphasized participants’ holistic stories of navigating reproductive care, here we center systemic mechanisms of racism and anti queerness embedded in health care delivery to extend and deepen that prior work. We can best understand the origins and complex issues surrounding racial, sex, and gender disparities affecting Black birthing individuals by examining the theoretical work of Black women scholars who have extensively studied race, gender, and reproduction [15–21]. The legacy of slavery and its connection to reproduction in the U.S. continues to influence the racial and gender power dynamics that create inequities and injustices in the lives and birthing experiences of Black individuals in healthcare [22, 23]. While concepts of obstetric violence [15] help highlight the inequities and violence faced by birthing people, the idea of obstetric racism serves as the primary framework for analyzing the roots of health and reproductive disparities among Black birthing individuals (regardless of their sexual orientation or gender and/or sex identity). Obstetric racism provides a lens to improve the assessment of reproductive health outcomes for Black birthing individuals [15].

Historically, reproductive health disparities among Black Queer birthing individuals have been examined through the lens of social conditions, including food and housing security, employment, education, and household responsibilities [17, 24]. Established theories of social determinants and intersectionality, such as the Sojourner Syndrome and the weathering hypothesis, reveal how racial and gender disparities, embodied trauma, and rates of birthing morbidity and mortality are consequences of institutional and obstetric racism as well as structural violence [25–27]. Obstetric racism is described as a distinct form of structural and interpersonal harm that is rooted in ongoing and historical dehumanization of Black birthing individuals in the healthcare system. It is a combination of neglect, abuse, coercion, and disregard that is directed at Black birthing people when navigating the healthcare system [15]. Obstetric racism builds upon centuries of reproductive control dating back to slavery, forced sterilization, and gynecological experimentation of enslaved Black women [15–19]. These reproductive health scholars focused on race, reproduction, and birthing health, caution against placing undue blame on Black birthing individuals for the consequences of social conditions and related adverse health outcomes.

This study aims to address the burdens faced by Black Queer birthing individuals by exploring their gestational-based lived experiences when navigating the healthcare system within the framework of obstetric racism. This framework helps illuminate why Black birthing people, especially those who are queer, experience disproportionately high rates of maternal mortality, lack of access to reproductive health care, and dismissal of their expertise and consent during labor. This approach is crucial not only for diagnosing contemporary health inequities but also for centering the lived realities of those most affected by obstetric harm. Thus, we investigate their health and social communication, language, and relationships, as well as the socioeconomic and historical medical dynamics at play that influence the interactions between Black Queer birthing individuals and medical providers. By understanding their perspectives, this research contributes to creating a roadmap for ensuring justice within the healthcare system.

## Methods

This paper presents a secondary thematic analysis of interviews previously collected as part of a larger qualitative health study on Black Queer birthing individuals’ reproductive health care experiences along their gestational journeys (e.g., miscarriage, abortion, pregnancy, conception, assisted reproductive technology, etc.). [3]. The original analysis used a storytelling approach grounded in Black feminist perspectives within a reproductive justice framework, emphasizing participants’ embodied storytelling as expertise and as counter-knowledge to dominant medical discourse. This framework shaped every stage of the research design, including:

1. Centering lived experience and storytelling as valid and necessary knowledge.
2. Recognizing intersecting oppressions racism, sexism, heteronormativity, transphobia, and structural barriers to reproductive care.
3. Foregrounding Black Queer epistemologies, resisting the assumption of white, heterosexual cisgender norms as default.
4. Emphasizing bodily autonomy and self-determination as fundamental reproductive justice principles.
5. Practicing reflexivity and accountability as researchers situated within systems of power.

These principles guided both the original study design and this reanalysis, which specifically reexamines the same transcripts through the lens of racism as a manifestation of structural oppression.

### Participants and recruitment

Ten Black Queer birthing individuals participated with an average age of 31 years. Participants self-identified with a range of sexualities (lesbian, queer lesbian, women-loving-women queer, queer and fluid, pansexual, pansexual bisexual, demisexual, bisexual, and another “just [research partner’s name]”) and gender identities (female, androgynous, gender fluid, spiritual being (as understood in Yoruba religion), non-binary, and non-binary/woman-presenting). All self-identified as Black or with African diasporic-related language (e.g., “African American,” “Black,” “Black Black,” “Cameroonian,” and “a Nigga Nigga”). The participants were recruited through social media outreach and reproductive justice networks. Inclusion criteria were: (1) self-identifying as Black and Queer and (2) having experienced at least one gestational experience (pregnancy, miscarriage, abortion, assisted reproductive technology, birth, etc.) in the U.S. A more detailed demographic table was included in the original publication [3]. To avoid duplication, we provide (Table 1) here a summarized participant overview relevant to the present re-analysis.

**Table 1:** Summary of participant demographic characteristics

*Categories*	*Identities Represented*	*Brief Definitions*
Sexual Identity	Queer lesbian; Women-loving-women queer; Queer fluid; Pansexual; Pansexual bisexual; Bisexual; Demisexual; Unlabeled (“just [name]”)	Participants described identities reflecting attraction to women, fluid or changing attraction, attraction across genders, or no sexual label.
Gender Identity	Female; Androgynous; Non-binary; Gender-fluid; non-binary woman-presenting; Spiritual being (Yoruba)	Identities included cisgender female, non-binary and gender-fluid expressions, and gender identities grounded in Yoruba cosmology.
Race/Ethnicity	African American; Black; “Black Black”; Cameroonian; “Nigga Nigga”	Participants used varied African diasporic and culturally situated terms of self-identification.

This table provides a condensed demographic overview to support transparency in this secondary analysis. Full, detailed identity-explication tables were published in the original study [3]

### Data collection

Semi-structured interviews were conducted virtually through Zoom, audio recorded, and transcribed verbatim. Interviews (or storytelling) lasted 45 to 120 min. Questions explore participants’ reproductive journeys, access to care, and experiences with the HCS. The University’s Institutional Review Board approved all procedures. Informed consents were provided after the pre-screening survey.

We conducted recruitment through Black Queer \-centered reproductive justice networks, community partners, and social media platforms, primarily Twitter/X and Instagram. Posts included short study descriptions, eligibility criteria, and contact instructions. All individuals who were interested in participating in the study completed a pre-screening survey to determine eligibility and, if eligible, were emailed to schedule interviews. All participants in this study received a $75 gift card. We addressed confidentiality by using encrypted communication and by allowing participants to use pseudonyms during interviews if preferred. A few participants faced digital access limitations, but to mitigate these barriers, we provided detailed instructions and guides for accessing the Zoom platform across multiple device types.

### Data analysis

For this paper, we re-read and re-coded all 10 transcripts to identify themes of obstetric racism and systemic injustice that were under-examined in the original publication. We engaged in iterative coding and peer debriefing to develop themes. Both authors coded data and met to resolve differences. This analytic lens differs from the original analysis, which focused on broader barriers to care; the current analysis specifically examines how systemic oppression manifests within participants’ stories.

Specifically, coding was conducted using NVivo 13. We used open coding to generate initial concepts, followed by focused coding to refine categories aligned with obstetric racism. Both authors maintained analytic memos, documented coding decisions in an audit trail, and engaged in scheduled peer debriefing meetings to review theme development. Discrepancies were resolved through discussion until consensus was achieved.

We selected a secondary thematic analysis approach because the original interviews were intentionally broad, narrative, and gestation-centered. This design created a rich dataset that allowed us to revisit participants’ stories through a different analytic lens, specifically, obstetric racism, without requiring new data collection. This approach is appropriate when existing data meaningfully address the new research questions and when further participant burden is ethically unnecessary.

By re-examining previously published interviews [3] through a broader lens, in conjunction with existing research on health disparities, we can see that their experiences reveal reproductive injustices that also affect others within this specific community and various identity-based groups. Moreover, by acknowledging the reproductive needs and desires of Black Queer birthing individuals through storytelling, we advocate for the reproductive healthcare needs of everyone, regardless of their social position. Lastly, the present analysis focuses on themes that were underexplored in the primary work, aiming to better elucidate partners’ experiences with obstetric racism and their recommendations for system changes from within.

### Ethical considerations

All participants in this study were informed in the original data collection’s consent materials that de-identified transcripts could be reanalyzed for research purposes beyond the initial study. For this secondary thematic analysis, all transcripts were re-reviewed and re-coded using pseudonyms and removal of location- or provider-identifying details, with all files stored on encrypted, access-restricted servers. Given the sensitivity of participants’ narratives, additional care was taken to avoid quoting material that could inadvertently reveal identity or specific reproductive events. Furthermore, as part of our commitment to justice-oriented research practices, all quotes used in the manuscript were shared with participants for their review.

## Results

In this section, we present the lived experiences of participants, Black Queer birthing people, which can be categorized into issues of healthcare access, healthcare support, and healthcare aftermath.

### Areas experienced as justice denied

#### Insurance and other health-related resources

##### Insurance

In the original analysis, it was determined that participants commonly described structural barriers to accessing care, particularly through the insurance companies and medical gatekeeping policies. In this secondary analysis, insurance emerged as a central site of obstetric racism, operating as an act of obstacle that undermines black queer birthing people’s attempts at family-forming. For example, a research partner who self-identified as Black and a nonbinary person (p7) mentioned:

“I sold my car to keep paying for IVF, and the insurance company kept telling me they couldn’t tell me what was covered until after I paid. It felt like they wanted me to fail.”

The storytelling above highlights the exclusionary insurance practices that compound with financial precarity. Many participants described how their sexual orientation and gender identity disqualified them from insurance coverage for assisted reproductive technology treatment. Most insurance policies mandate a medical infertility diagnosis and require attempts to conceive with an opposite-sex partner for coverage [28]. This disregards social infertility, where individuals need assisted reproductive technology (ART) due to circumstances like sexual and gender diversity or becoming single parents by choice [29].

The challenges are even more pronounced for Black Queer individuals, nearly half of whom may find ART inaccessible due to economic barriers; 40% live below the poverty line [2], and ART costs can average between 15,000 per cycle [30]. P7’s story emphasizes that one of the most difficult parts of their IVF journey was navigating the insurance providers who offered little guidance or informational support on the coverage eligibility of their assisted reproductive technology-based care. Research indicates that over half of families struggle with the financial burden of ART, particularly those with low incomes, long after the treatment is initiated [31]. These financial and coverage disparities impinge on the reproductive healthcare rights of Black Queer people, who face compounded structural racial and gender-based discrimination. Discussing healthcare access necessitates a focus on health insurance, particularly for Black queer individuals in the U.S. Future researchers and health providers should focus on the distinct challenges they face in accessing ART and advocate for policies that improve access to reproductive resources for Black Queer birthing individuals.

##### Other health-related resources

Despite Black Queer birthing people having similar rates of child-rearing as their non-2SLGBTQIA + counterparts [32], they experience higher levels of food insecurity, unemployment, and limited healthcare access [2]. Many rely on Medicaid, which, while slowly improving for the Black people living in the U.S, does not cover essential expenses like housing [2]. Housing security is crucial for overall well-being, yet many assistance programs are underfunded, leaving 75% of eligible households without support [33]. A participant (p6) reported that they experienced homelessness shortly after experiencing a high-risk birth and giving birth to their premature child due to their need to be away from work because of spending extensive time at the hospital healing.

“We lost our apartment with my second child because […] I ended up being on bed rest for a month and a half in the hospital, where I couldn’t work, so we couldn’t pay rent […] When I came home from the hospital. We ended up moving in with my mom, who had moved back into town because I couldn’t afford our rent.”

Navigating the complex healthcare system can cause significant stress and confusion, particularly for uninsured Black Queer individuals who may struggle to achieve the health literacy necessary for effective navigation [34]. This often leads to delayed care or avoidance of medical costs, worsening health disparities [34]. Future researchers and healthcare providers should focus on the unique challenges faced by Black Queer birthing individuals in accessing health benefits and inquire about their potential challenges beyond health-related concerns.

#### Lack of support throughout the process

##### Encountering racism and anti-queerness during reproduction

The gestational-based stories that we shared by Black Queer birthing people revealed the ways that anti-queer values influence institutional practices, leading to misunderstandings about relationships, such as assuming a partner’s gender based on titles like “fiancé” or visual cues instead of prioritizing relationship building with patients that centers on getting to know them and their needs. For example, one lesbian identified participant (p5) mentioned:“

“I went to the [fertility]…looking back on it, I really didn’t like it, because they was constantly telling me like, oh well, when are you and your husband… and I was like my fiance she’s a woman.”

These instances of misconstructions of gender expression reinforce barriers for Queer individuals seeking fertility care, particularly because it prevents Black Queer birthing people from getting the care that they deserve, emphasizing the need for inclusive language and access via understanding of gender-affirming care to ART services beyond traditional notions of infertility for heterosexual couples [9]. Most US insurance policies mandate a medical infertility diagnosis and require proof of attempts at conception with an opposite sex partner for coverage for at least six months [29]. These policies disregard “social infertility,” where individuals seek assisted reproductive technology services due to sexual and gender diversity or as single parents by choice.

Additionally, a participant (p4) spoke to the economic barriers and racial disparities in the ART process due to the lack of Black sperm donor availability.

“Really like trying to find a black donor. Because you know we started to notice that, like a lot of the banks that they had didn’t have a variety of like black men. […] then we did research you read that you know, sometimes black men don’t like to donate their sperm, and it was like you know either we’re going to find a black donor or we’re not going to have no kid. We need a black child.”

This lived experience of the participant echoes the racial disparities that are reflected in national data on access to sperm, particularly for Black individuals, with only 2% of donors in major U.S. sperm banks being Black [35]. There is minimal investment from the health community in addressing this shortage, which limits reproductive choices [36]. Outdated policies, such as excluding gay men from sperm donation, alongside genealogical requirements that disadvantage Black people (such as requiring three to five generations of family history), further exacerbate these issues [35, 37]. The high costs of ART often lead Queer individuals to prefer at-home insemination methods [38]. Thus, these increased social and financial barriers limit Black and Queer birthing people from having the *choice* they deserve in their ART-based decision making, such as receiving support from the HCS.

##### Birthing rights ignored by providers

Participants also reported that medical providers failed to recognize their expertise regarding their bodies, particularly during childbirth, and this was a common theme identified in the original analysis. However, re-analysis also finds racialized components of this experience. One of the participants (p6) described A traumatic labor intervention where the doctor manually ruptured their membranes during labor without their consent. Another participant (p3) mentioned telling their doctor before labor:

“Hey, we want you to talk to us before you do anything. Let us know you know what’s about to happen. We are a part of this process, right? But the nurse looked at my partner. It was just like he’s the doctor. They said he’s the doctor like. How dare you want them to explain?”

Research highlights a common theme of Black birthing people experiencing a lack of bodily autonomy and their preferences being overlooked by healthcare providers [13]. A California Health Report survey found that Black birthing people’s preferences are less likely to be heard compared to those of other racial/ethnic groups [13]. A more recent study of pregnant Black individuals revealed a desire for collaborative care that meets their unique needs, yet many still faced inequitable treatment [4]. To address these disparities, healthcare providers must take a justice-oriented approach [21] that acknowledges that birthing people, regardless of race, sexuality, or gender, are the experts in their own health and should have the autonomy to make their own reproductive care decisions.

##### Little post-experience support

Black Queer individuals face unique challenges within the HCS, which increase their risk of severe mental illnesses. One in four Black Queer people will be diagnosed with depression, and 82% experience daily discrimination [2], further compounding their mental health struggles, including during pregnancy, pregnancy termination, and postpartum. Research on the mental health of Black mothers, particularly those who are Queer, is insufficient, especially concerning postpartum depression and anxiety. The lack of literature on Black Queer birthing experiences makes it difficult to understand the health challenges they face during periods of heightened mental strain, such as pregnancy, abortion, or miscarriage. Many research partners reported unmet mental health care needs due to stigma and access issues. Future researchers and medical providers should focus on connecting patients with available mental health services and consider the specific needs of Black Queer birthing individuals and how providers communicate about mental health care along gestational journeys.

Additionally, the aftermath of pregnancy termination and the impact it has on mental health was a major theme experienced by our research partners, yet it is rarely addressed, even by those who champion abortion rights. Research partners described their abortion experiences as ongoing journeys that influence their emotions and sense of disconnection from their bodies. Black Queer individuals face compounded feelings of isolation and shame due to societal stigma around their sexuality and reproductive choices. Queer people in the U.S. seek abortion services at higher rates than heterosexual individuals [39], with Black birthing people specifically seeking care more often [40]. While abortion stigma is well-documented, there’s a lack of discussion on the intensified stigma Black Queer people face due to their intersecting lived identities. The foundation of abortion stigma revolves around moral objections and societal beliefs about reproduction, closely tied to anti-queerness. Consequently, Black Queer birthing people encounter heightened shame due to their race, sexuality, and health behaviors.

Abortion clinics and health researchers should prioritize the experiences and storytelling of Black Queer individuals to enhance understanding of the stigma surrounding abortion, as the impacts of abortion are not time-bound but result in ongoing emotional and physiological effects. Planned Parenthood addresses post-abortion changes separately, instead of interconnected, and should be considered together. For example, their website states [41]:

“Feelings of relief, sadness, elation, or depression are common and may be strong due to the hormonal changes that occur after an abortion. Most people find these feelings do not last very long (emphasized).”

The emotional changes after abortion, as described by Planned Parenthood [41], oversimplify the complex relationship between birthing people’s emotions and their gestational bodies. A research partner reported that their emotional and physical experiences are interconnected, with them experiencing feelings of guilt and self-doubt post-procedure. This connection affects their future health decisions and challenges the mind-body split often perpetuated in healthcare, which assumes psychological health is separate from physical health. It’s crucial for health providers to address this phenomenon, especially for Black Queer birthing people, to foster more holistic health insights and improve the quality of health information provided.

### Moving toward justice in the HCS and toward justice: learning from community-based reproductive health spaces

While advocating for systemic change in traditional healthcare, it is essential to acknowledge the efforts of advocates in alternative spaces that help mitigate issues within the healthcare system. Many individuals find autonomy and safe spaces with community-based doulas and midwives [4, 42]. For instance, one research partner sought doula care services because they could not find supportive healthcare providers who understood their identity as a fat Black Queer disabled birthing person. Unfortunately, the healthcare system often dismisses the benefits of community-based reproductive health support. For example, health insurance rarely covers community-based doulas and midwives, and there is a lack of representation of community-based doulas and midwives in traditional HCS settings [42].

When exploring the storytelling of one research partner’s pregnancy journey, they detail asking their doctor in the hospital how he felt about “natural birth”, referring to their desire for unmedicated labor. They describe the response as, “Everything natural is not good. Hurricanes are natural, earthquakes are natural. Cancer can be natural. I also cried.” Unfortunately, for this partner, their desire to have an unmedicated birth was mocked by the doctor and compared to experiencing severe, life-threatening illnesses and weather-related conditions. This partner’s experience underscores a broader issue where one is criticized for deviating from traditional healthcare practices. In contrast, community-based doulas and midwives often employ communication techniques that foster trust and emphasize concepts such relational autonomy [43, 44], free from coercion, which honor patient’s multiple intersecting identities and lived experiences when guiding them in making reproductive health decisions. To the extent that the HCS and community-based spaces can coexist and coordinate, some of the practices of community health professionals could be incorporated into the HCS context.

## Discussion

This paper contributes to the literature as a critical review of reproductive injustices informed by a secondary thematic analysis of interviews with Black Queer birthing individuals.

Whereas previous publications centered their full gestational-based storytelling [3], this paper focused specifically on systemic patterns obstetric racism within those stories. Our analysis of Black Queer birthing people’s experiences with navigating the fertility- and pregnancy-related healthcare system from a lens of places and spaces where justice is most often denied can elucidate opportunities for systemic reform that benefit all who seek healthcare access. Our research partners indicated that the points of contact with the healthcare system during their infertility, pregnancy, and/or termination journeys that were particularly challenging were in the spaces of insurance, navigating other ancillary needs such as housing, barriers in seeking care and autonomy during childbirth, and the lack of supports for mental and physical health after acute care is over. While these findings corroborate existing literatures on the ways the healthcare system contributes to worse outcomes for Black and Queer populations compared to their White heterosexual counterparts [2, 7], they also add nuance and individual idiosyncrasies to what it means to experience those negative outcomes directly, and what the people most affected by these broader societal patterns see as the causes and opportunities involved.

These findings further substantiate the growing critique that obstetric racism is not an anomaly but a normative function of the reproductive healthcare system as it exists in the United States. Drawing on critical Black feminist scholarship and reproductive justice frameworks, our re-analysis illustrates how obstetric racism operates both through overt acts, such as consensual procedures, and through systemic design, including insurance gatekeeping and racial biases in ART access [17, 19, 21]. By centering the stories of Black Queer birthing people, we reaffirm that addressing reproductive injustices requires more than cultural competency or anti-bias training; it demands systemic transformation grounded in an understanding of how racism, anti-queerness, and medical neglect are embedded in the logics of healthcare delivery itself.

Crucially, not only do barriers within the healthcare system that negatively impact the physical and mental health of this population need to be urgently addressed, but our partners also provided a new avenue for improvement by embracing community-based care systems and holistic medicine, such as that provided by doulas, midwives, and birthing centers. These resources have the potential to help fill some of the gaps left by the traditional healthcare system, which perpetuates the injustices experienced by our research partners. That said, we do not suggest that these alternative resources can counteract the negatives of the traditional system, but that their inclusion during the process of restructuring within the system can potentially help people to navigate and circumvent some of those barriers.

This study has several limitations. As a secondary analysis of small, purposively selected sample, findings are not generalizable. The scope of this analysis was shaped by the original interview design and our interpretive lens as researchers. Our focus on systemic patterns being participant voices are used illustratively rather than exhaustively calling the full richness of their narratives is reported in our prior publication period future work should center participant stories more fully and engage larger and more diverse samples of Black Queer birthing people. However, given the significant gap in reproductive health research centering Black Queer birthing people, this study contributes to an emerging and necessary area of scholarship.

## Conclusion

Black Queer birthing people experience disproportionately poor outcomes in reproductive healthcare due to compounding systemic inequities and discrimination. This paper presents a critical literature review enhanced by secondary qualitative analysis that offers insights on how the healthcare system can better support Black Queer birthing people. Additionally, we detail how more dialogue and research are needed to partner with Black Queer people to ensure equitable access, treatment, and services, thereby improving reproductive justice for all.

## Data Availability

Not applicable.
